# Relationship between mental disorders and non-traumatic cerebral hemorrhage: cross-sectional analysis and mendelian randomization

**DOI:** 10.7717/peerj.21385

**Published:** 2026-06-29

**Authors:** Fangqi Hu, Yunsong Pan, Ningning Lu, Jie He, Rui Zhang, Xinxu Wu, Tianpeng Zhang, Hui Zhou, Hui Shi

**Affiliations:** 1 Department of Neurosurgery, Lianyungang Clinical College of Nanjing Medical University/ The First People’s Hospital of Lianyungang, Lianyungang, Jiangsu, China; 2 The Affiliated Lianyungang Hospital of Xuzhou Medical University/ The First People’s Hospital of Lianyungang, Lianyungang, Jiangsu, China; 3 Kangda College of Nanjing Medical University, Lianyungang, Jiangsu, China

**Keywords:** Dementia, Non-traumatic cerebral hemorrhage, MIMIC, Mendelian randomization, Mental disorders

## Abstract

**Background:**

The causality between mental disorders and spontaneous non-traumatic cerebral hemorrhage (NCH) has not been sufficiently explored. This study aims to investigate the associations of a broad spectrum of 16 mental disorders with NCH through Mendelian randomization (MR) analysis and to assess the association in a real-world study.

**Methods:**

Univariate analysis and mediational MR analysis were employed to investigate the potential association between mental disorders and NCH. Genetic instruments associated with mental disorders were identified through a genome-wide association study (GWAS) using the FinnGen database of European populations. Furthermore, the MR results were corroborated based on the real population (MIMIC-IV database). The correlation between mental disorders and the risk of NCH was examined using logistic regression models, which progressively added confounding factors for adjustments.

**Results:**

Univariate MR analysis showed that genetically predicted dementia was significantly associated with NCH (OR = 1.090, 95% CI [1.049–1.133], *p* = 1.16 × 10^−5^); However, no statistically significant associations were observed between NCH and other mental disorders, including attention-deficit/hyperactivity disorder, Lewy body dementia, depression, eating disorders, gender identity disorder, habit and impulse disorders, obsessive-compulsive disorder, persistent mood disorders, phobic anxiety disorders, post-traumatic stress disorder, sleep disorders, somatoform disorders, anxiety disorders, autism spectrum disorders, and bipolar disorder. Reverse MR analysis further indicated that there is no reverse causal relationship between dementia and NCH. When several confounding factors were taken into account, observational studies revealed that dementia was linked to an increased risk of NCH (OR = 1.31, 95% CI [1.07–1.59], *p* = 0.007). Additionally, we did not observe any intermediate factors that can mediate dementia and NCH.

**Conclusions:**

Results of both MR analyses and observational studies showed that dementia was associated with the risk of NCH. Furthermore, reverse MR analysis revealed no causal effect of NCH on dementia.

## Introduction

Globally, mental disorders are a leading cause of disability, accounting for 13% of the burden of disease (Buka, 2008; Eaton et al., 2008). Such diseases not only result in huge direct medical expenses but also lead to increased demand for social services and family care burdens through lost productivity (Arias, Saxena & Verguet, 2022; Collaborators, 2022). At the individual level, people with mental disorders often face multiple challenges, including impaired social functioning, reduced quality of life, and shortened life expectancy (Nunes & Da Rocha, 2022).

Non-traumatic cerebral hemorrhage (NCH), the second most common subtype of stroke, is linked to a high rate of death and morbidity worldwide (Feigin et al., 2009; Van Asch et al., 2010). Studies have shown that lobar hemorrhage is different from deep cerebral hemorrhage in terms of pathogenesis, clinical manifestations, and prognostic features. Its clinical manifestations are more significant neurological deficits and poor prognosis (Mendiola et al., 2023). Current evidence suggests that, in addition to traumatic factors, non-traumatic mechanisms such as neuroinflammation, tryptophan metabolism abnormalities, hypertension-related vascular factors, and brain oxidative stress also contribute to the pathogenesis of cerebrovascular diseases, neurological disorders, and mental disorders, and these factors may interact synergistically (Huang et al., 2023; Wang, Xiong & Tang, 2017). Determining whether a mental disorder precedes or follows a cerebrovascular disease is often challenging due to shared pathogenic factors. However, the association between mental disorders and the risk of NCH requires further research to support.

Previous research has explored the temporal correlation between mental disorders and NCH, suggesting that mental disorders may precede cerebrovascular events, but the causal direction remains to be fully elucidated. MR is a causal inference method based on genetic variation that can use instrumental variables to indirectly assess the potential causal relationship between exposure factors and outcomes (Sekula et al., 2016). The utilized genetic variants, which are unaffected by the onset and course of the disease, are discovered at the fetal stage (Verduijn et al., 2010). It is suitable for determining the causality between mental disorders and NCH that share common pathogenic factors. This study aims to assess the connections between mental disorders and NCH through MR analyses. Additionally, the reverse MR analyses were performed to improve the reliability of causal inference and further avoid reverse causality.

## Methods

### Mendelian randomization analysis

#### Selection of genetic instruments

Three basic assumptions are required for genetic variations to be classified as effective instrumental variables (IV): (1) They should be closely connected with exposure; (2) they should not be associated with any potential confounding factors related to expose-outcome; and (3) They should not affect the results through any other variables other than exposure (Hemani et al., 2018). An independent single-nucleotide polymorphism (SNP) was utilized to verify the initial MR hypothesis. When conducting forward MR analyses, given the limited size of the selected databases, we relaxed the selection thresholds to obtain a sufficient number of instrumental variables. Specifically, the inclusion criteria for instrumental variables were set as follows: significant genome-wide association with the exposure factor (*p* < 5 ×10^−^^6^), and no strong linkage disequilibrium (*r*^2^ < 0.001) between variables within a 10,000-kilobase genomic region; In reverse MR analysis, the selected instrumental variables must achieve a significant association level with the exposure factor (*p* < 5 × 10^−^^8^), while maintaining the same linkage disequilibrium standard (*r*^2^ < 0.001) and genome window size (10,000 kb). We used the *F* statistic (*β*^2^/SE^2^) to evaluate the strength of the instrumental variables and calculated the percentage of phenotypic variation interpreted by the entire SNP set in order to improve the initial hypothesis. A powerful instrument was defined as having an F-statistic greater than 10 (Burgess, Thompson & Collaboration, 2011). The genetic variability interpreted by each SNP was determined, and *R*^2^ was determined as *β*^2^/(*β*^2 ^+ SE^2^*(*N* − 2)), where *β* is the effect size, SE is the standard error, and N is the sample size (Tian et al., 2022) (Table S1).

#### Data source

Our main source was the Genome-Wide Association Study (GWAS) database, which provides publicly accessible summary statistics on populations of European ancestry. All studies have received current ethical clearances from institutional review boards separately. As all analyses involved in this study were based on publicly available summary statistics, it did not require ethical approval from an institutional review board.

Exposure included 16 different mental disorders: attention deficit hyperactivity disorder, dementia with Lewy bodies, depression, eating disorders, gender identity disorders, habit and impulse disorders (IDs), obsessive-compulsive disorder (OCD), persistent mood disorders, phobic anxiety disorders, post-traumatic stress disorder (PTSD), sleep disorders, somatoform disorder, anxiety disorders, dementia, autism spectrum disorder, and bipolar disorder. Exposed data were sourced from FinnGen (https://www.finngen.fi/fi) and the MRC-IEU online database (https://gwas.mrcieu.ac.uk/). In the MRC-IEU database, since they come from different research teams, the analytical methods used and the confounding factors controlled for are not entirely consistent (Chia et al., 2021; Dönertaş et al., 2021; Dönertaş et al., 2021; Elsworth et al., 2020; Martin et al., 2018). Individuals of unknown gender, with a higher missing rate of genotype (>5%), severe heterozygosity (± 4 standard deviations (SDs)), and non-Finnish ancestry were removed from FinnGen.

A total of 21 potential intermediate factors were selected according to the existing studies and reviews, including blood platelet count (PLT) (Al-Shahi Salman & Greenberg, 2023), tight junction protein zonula occludens-1 (TJP-ZO-1) (Lecca et al., 2023), fibrinogen (McBride, Tang & Zhang, 2017), D-dimer (Johansson et al., 2018), C-reactive protein (CRP) (Myserlis, Anderson & Georgakis, 2023), tumor necrosis factor (TNF) (Wang et al., 2018), endothelin (ET) (Suhardja, 2004), endothelin-converting enzyme (ECE) (Kuruppu et al., 2014), brain-derived neurotrophic factor (BDNF) (Hwang et al., 2013), cerebral dopamine neurotrophic factor (CDNF) (Zhang et al., 2018), low-density lipoprotein (LDL) (Cheng et al., 2020), high-density lipoprotein (HDL) (Wang et al., 2011), total cholesterol (TC) (Ma et al., 2016), apolipoprotein A (APO-A), apolipoprotein B (APO-B), apolipoprotein E (APO-E), apolipoprotein B/apolipoprotein A1 (APO-B/APO-A1) (Yu et al., 2022), interleukin-6 (IL6) (Leasure et al., 2021), hemoglobin (HG) (Roh et al., 2019) and tau protein (Hu et al., 2012). GWAS data of potential intermediate factors were based on cohorts of European ancestry or primarily European ancestry. (1) Mental disorders should be causally related to intermediate factors with an effect size of Beta (XZ); (2) Intermediate factors should be causally related to NCH with an effect size of Beta (ZY); (3) The mediating effect Beta (XY) was calculated as Beta (XZ) × Beta (ZY). The testing required two separate steps: (1) To confirm whether Beta (XZ) and Beta (ZY) are significant; If one of them is not significant, the test would be interrupted. (2) To verify whether Beta (XY) is significant; It should be considered as a complete mediation if not significant and as an incomplete mediation if significant (Carter et al., 2021). When selecting instrumental variables for PLT and TJP-ZO-1, the limited number of instrumental variables reaching statistical significance (*p* < 1.00 × 10^−^^8^) may result in weak instrumental variable bias (Sanderson et al., 2022). Therefore, we referenced previous studies (Wen et al., 2024) and relaxed the screening thresholds: significant level (*p* < 1.00 × 10^−^^6^) and independent linkage disequilibrium level (LD *r*^2^ < 0.001, with the linkage disequilibrium range within 10,000 kb). Other intermediate factors were screened based on the significant level of *p* < 1.00 × 10^−^^8^ and an independent level of LD *r*^2^ < 0.001 within 10,000 kb.

The outcome measures of NCH (ID: finngen_R9_I9_INTRACRA) were sourced from the FinnGen database, including 205,862 Europeans (2,794 patients and 203,068 individuals from the control group) with 16,380,408 SNPs.

#### Statistical analysis

In this study, we adopted the inverse-variance weighting (IVW) method to combine the effect size of each IV (Thilo et al., 2011). As supplemental measures for IVW, MR-Egger and weighted median were employed. To assess the heterogeneity, the Cochran’s Q value was utilized. An MR-Egger intercept was employed to detect the horizontal pleiotropy. Moreover, reverse MR analyses were conducted. The IVW model was primarily applied in reverse MR analyses with MR-Egger and weighted median as the complementary (Veres et al., 2015). Statistical analyses were performed using R software (Version 4.3.3). The threshold for statistical significance was set at *p* < 0.05.

### Observational epidemiologic study

#### Data source

The Medical Information Mart for Intensive Care IV (MIMIC-IV) database, a comprehensive single-center resource run by the Massachusetts Institute of Technology (MIT), provided the raw data. Patient information from Beth Israel Deaconess Medical Center (BIDMC) admissions from 2008 to 2019 is included in this database (Chia et al., 2021). Both the MIT and BIDMC Institutional Review Boards have approved the MIMIC database, and all patient data has been anonymised.

#### Study population

This study included the population in the MIMIC-IV database (version 2.2). Exclusion criteria: (1) age ≤ 18 years; (2) missing follow-up data; (3) missing international disease diagnosis codes. Mental disorders and NCH are defined by ICD-9 as well as ICD-10. Since the time of onset of NCH could not be determined in the MIMIC-IV database, a cross-sectional design was adopted in this study. Only data from the first admission were analyzed if the patient had been admitted more than once. Several confounding factors were excluded from the MIMIC-IV database’s data for every patient in this investigation. These include age, gender, hypertension, cerebral aneurysms, alcohol use, nicotine dependence, coagulation disorders, creatine kinase isoenzyme MB (CKMB), activated partial thromboplastin time (APTT), and blood platelet count (PLT). For laboratory data with multiple measurements, we only analyzed those measured at the first time. Furthermore, to avoid bias caused by sample size loss, for variables with missing values less than 25%, a multiple imputation method based on random forest was applied to interpolate missing data. Multiple interpolation employs the missForest package with default parameters: The maximum iteration count (maxiter) is set to 10, and the number of trees (ntree) is set to 100. For variables with a missing rate exceeding 25%, we encode the missing state as a separate category. Among these, the missing rate for platelets, PT, and PTT is <25%; the missing rate for CKMB is >25%.

#### Statistical analysis

To estimate odds ratios (ORs) and 95% confidence intervals (CIs), multivariable logistic regression models were used. Models were progressively modified: Model 1: Unadjusted; Model 2: Age- and gender-based adjustments; Model 3: Adjusted based on age, gender, hypertension, cerebral aneurysms, alcohol use, nicotine dependence, coagulation disorders, CKMB, APTT, and PLT. Statistical analyses were performed using R software (Version 4.3.3). To control for Type I errors resulting from multiple comparisons, the results of multiple tests were adjusted using the Bonferroni correction. The threshold for statistical significance was set at *p* < 0.05.

## Results

### Mendelian randomization

**Figure 1:**
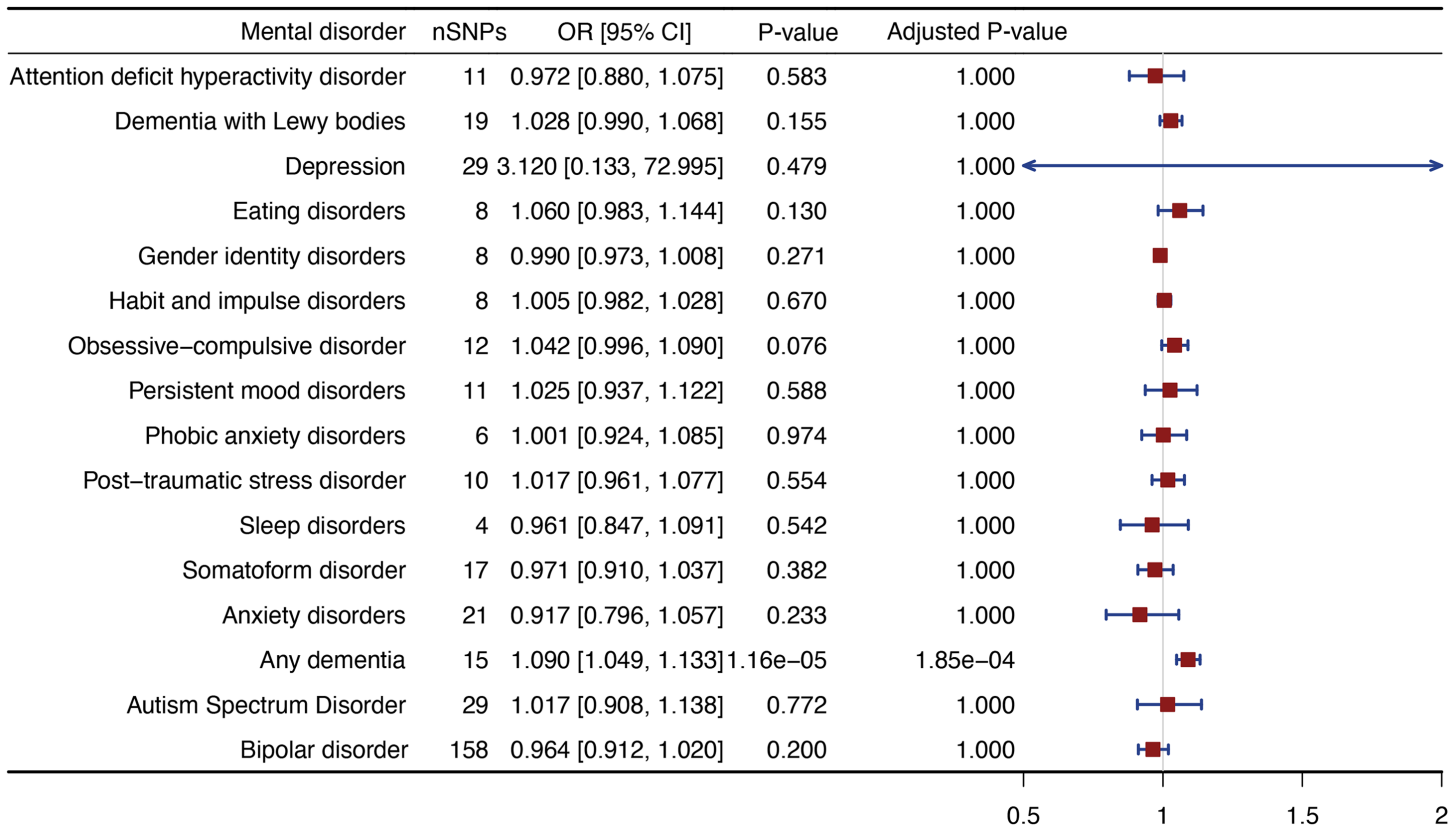
Results of univariate analysis of Mendelian randomization (inverse variance weighting). SNP, single nucleotide polymorphism; OR, odds ratio; CI, confidence interval.

The univariate MR analyses demonstrated that the genetically predicted dementia (OR = 1.090, 95% CI [1.049–1.133], *p* = 1.16  × 10^−^^5^, adjustied *p* = 1.85e ×10^−4^) was causally related to NCH. Additionally, this association is consistent with the findings of the MR-Egger approach (OR = 1.073, 95% CI [1.019–1.130], *p* = 0.0199) and weighted median (OR = 1.080, 95% CI [1.036–1.126], *p* = 0.0003) (Table S2). Moreover, no causal relationship was found between NCH and other mental disorders (*e.g.*, attention deficit hyperactivity disorder, dementia with Lewy bodies, depression, eating disorders, gender identity disorders, IDs, OCD, persistent mood disorders, phobic anxiety disorders, PTSD, sleep disorders, somatoform disorder, anxiety disorders, autism spectrum disorder, and bipolar disorder) (Fig. 1).

Since NCH affects the function of brain regions, we conducted a reverse MR analysis of dementia and NCH. The results showed that NCH had no significant effect on the risk of developing dementia (OR = 0.958, 95% CI [0.884–1.038]; *p* = 0.290), and no pleiotropy (*p*-value of Egger intercept: 0.4125) or heterogeneity (*Q* = 11.574, Q *p*-value = 0.5628) was observed (Table S3).

To explore the potential intermediate factors underlying the association between dementia and non-traumatic cerebral hemorrhage (NCH), we included possible risk factors for NCH, including PLT (Al-Shahi Salman & Greenberg, 2023), TJP-ZO-1 (Lecca et al., 2023), fibrinogen (McBride, Tang & Zhang, 2017), D-dimer (Johansson et al., 2018), CRP (Myserlis, Anderson & Georgakis, 2023), TNF (Wang et al., 2018), ET (Suhardja, 2004), ECE (Kuruppu et al., 2014), BDNF (Hwang et al., 2013), CDNF (Zhang et al., 2018), LDL (Cheng et al., 2020), HDL (Wang et al., 2011), TC (Ma et al., 2016), APO-A, APO-B, APO-E, APO-B/APO-A1 (Yu et al., 2022), IL6 (Leasure et al., 2021), HG (Roh et al., 2019) and tau protein (Hu et al., 2012). Eligible mediating factors were identified in accordance with established criteria.

During the first phase of the mediation analysis (Dementia ->Mediator), genetically predicted dementia showed significant causal effects on several candidate mediators, including APO-B (OR = 1.216, 95% CI [1.140–1.296], *p* = 2.606  × 10^−^^9^), APO-E (OR = 1.212, 95% CI [1.138–1.291], *p* = 2.071 × 10^−^^9^), LDL (OR = 1.207, 95% CI [1.137–1.282], *p* = 8.743  × 10^−^^10^), APO-B/APO-A1 (OR = 1.155, 95% CI [1.098–1.215], *p* = 2.449  × 10^−^^8^), TC (OR = 1.139, 95% CI [1.079–1.202], *p* = 2.374  × 10^−^^6^), TG (OR = 1.043, 95% CI [1.025–1.060], *p* = 9.648  × 10^−^^7^), TJP-ZO-1 (OR = 1.166, 95% CI [1.099–1.238], *p* = 4.533  × 10^−^^7^), APO-A (OR = 0.925, 95% CI [0.897–0.954], *p* = 8.723  × 10^−^^7^), HDL (OR = 0.949, 95% CI [0.924–0.975], *p* = 1.16  × 10^−^^5^), and PLT (OR = 0.980, 95% CI [0.969–0.992], *p* = 7.00  × 10^−^^4^).

**Figure 2:**
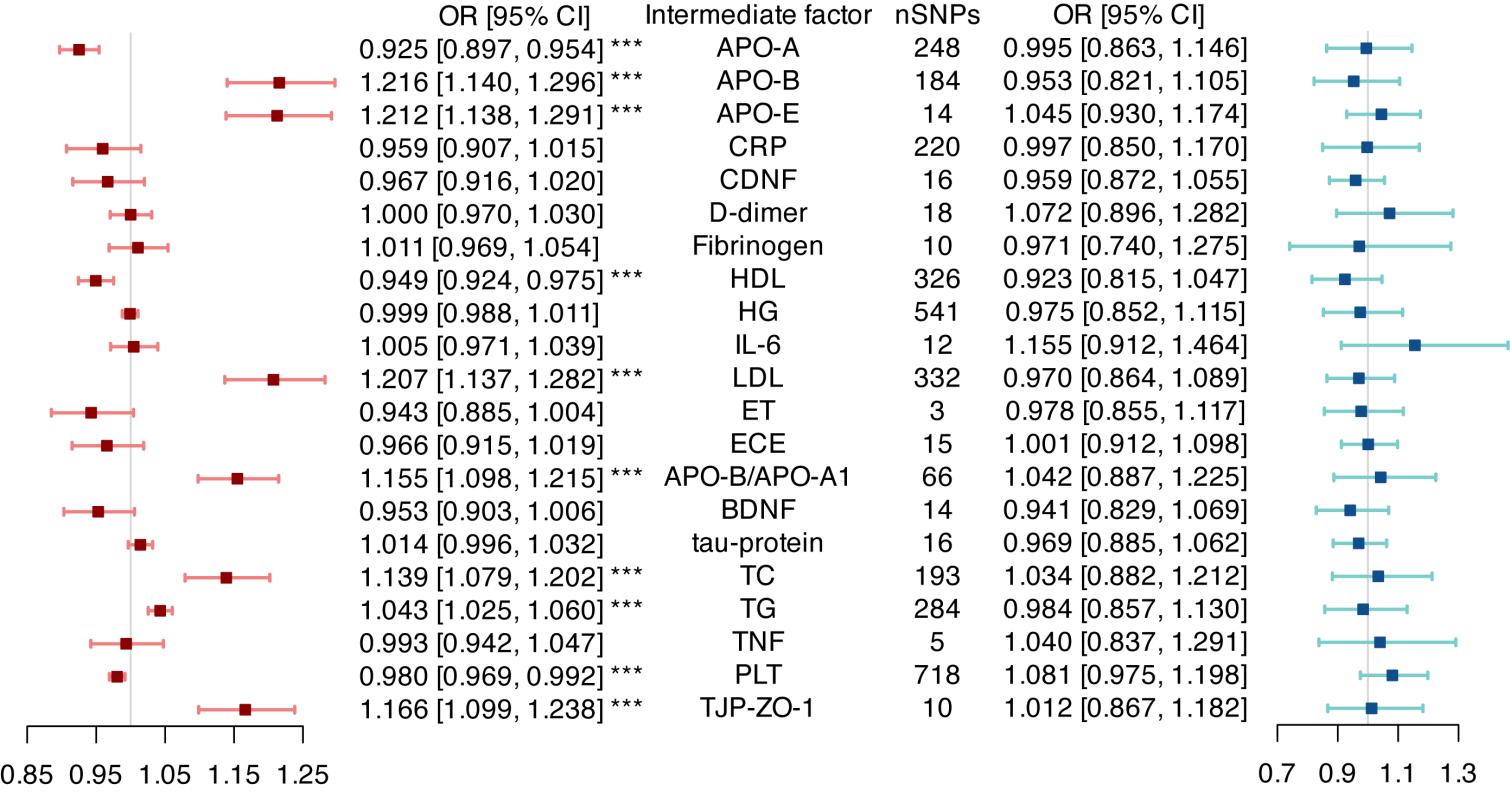
Results of Mendelian randomized mediation analysis (inverse variance weighted). The red line segments represent the results of dementia and intermediate factors, and the blue line segments represent the results of intermediate factors and NCH. *p < 0.05; **p < 0.01; ***p < 0.001. SNP, single nucleotide polymorphism; NCH, n on non -traumatic cerebral hemorrhage; APO, apolipoprotein; CRP, C-reactive protein; CDNF, cerebral dopamine neurotrophic factor; HDL, high-density lipoprotein; BDNF, brain-derived neurotrophic factor; LDL, low-density lipoprotein; TC, total cholesterol; IL, interleukin; TNF, tumor necrosis factor; ET, endothelin; ECE, endothelin-converting enzyme; PLT, platelet count; TJP-ZO-1, tight junction protein ZO-1.

However, in the second phase (Mediator → NCH), none of these candidate mediators showed evidence of a causal association with NCH. Therefore, although dementia exerted causal effects on several candidate mediators in the first step, no complete mediation pathway was supported in the subsequent analysis (Fig. 2, Table S4).

### Observational epidemiologic study

Finally, this study included 180,733 patients from the MIMIC database. Among them, 3,403 patients had NCH. A sensitivity analysis was conducted on the data before and after multiple interpolations. The results indicate that there is no significant difference in the variables before and after interpolation (see Table S5). Table 1 provides specifics about the baseline characteristics of patients. Patients with NCH had a higher prevalence of cerebral aneurysms (*p* < 0.001), coagulation disorders (*p* < 0.001), dementia (*p* < 0.001), hypertension (*p* < 0.001), and respiratory failure (*p* < 0.001). NCH was more likely to occur in men (*p* < 0.001), alcohol drinkers (*p* = 0.004), and older adults (*p* < 0.001). Platelet counts and PTT were relatively lower in patients with NCH (*p* < 0.001).

**Table 1:** Baseline characteristics of patients.

**Characteristic**	**Overall**, *N* = 180,733^1^	Without NCH, *N* = 177,330^1^	NCH, *N* = 3,403^1^	***p*-value** ^2^
Alcohol	7,499 (4.1%)	7,325 (4.1%)	174 (5.1%)	0.004^**^
Cerebral aneurysm	1,301 (0.7%)	1,178 (0.7%)	123 (3.6%)	<0.001^***^
Coagulation deficiencies	10,575 (5.9%)	10,203 (5.8%)	372 (11%)	<0.001^***^
Dementia	2,761 (1.5%)	2,649 (1.5%)	112 (3.3%)	<0.001^***^
Hypertension	62,525 (35%)	60,544 (34%)	1,981 (58%)	<0.001^***^
Nicotine dependence	6,527 (3.6%)	6,405 (3.6%)	122 (3.6%)	>0.9
Respiratory failure	7,751 (4.3%)	7,139 (4.0%)	612 (18%)	<0.001^***^
Gender				<0.001^***^
Female	95,729 (53%)	94,091 (53%)	1,638 (48%)	
Male	85,004 (47%)	83,239 (47%)	1,765 (52%)	
Age				<0.001^***^
≤60 years	102,245 (57%)	101,114 (57%)	1,131 (33%)	
>60 years	78,488 (43%)	76,216 (43%)	2,272 (67%)	
CKMB				0.4
≤ 10 ng/mL	43,975 (87%)	42,582 (87%)	1,393 (87%)	
>10 ng/mL	6,388 (13%)	6,174 (13%)	214 (13%)	
Missing	130,370	128,574	1,796	
Platelets (10ˆ9/L)	244 [196, 295]	245 [197, 296]	222 [177, 274]	<0.001^***^
PTT (s)	29 [27, 32]	29 [27, 32]	28 [25, 31]	<0.001^***^

**Notes.**

CKMB creatine kinase isoenzyme PTT partial thromboplastin time NCH nontraumatic cerebral hemorrhage

1 n (%); Median [IQR]

2 *p < 0.05; **p < 0.01; ***p < 0.001.

Statistical analysis: Continuous variables are presented as median (IQR) and were compared using the Mann–Whitney U test. Categorical variables are presented as n (%) and were compared using the Chi-square test.

Subsequently, we employed a logistic regression model to investigate the relationship between Mental Disorders and NCH in the real population, and gradually incorporated confounding factors for adjustments (Model 1: Unadjusted; Model 2: Age- and gender-based adjustments; Model 3: Adjusted based on age, gender, hypertension, cerebral aneurysms, alcohol use, nicotine dependence, coagulation disorders, CKMB, APTT, and PLT). The analysis results showed that, among unadjusted (OR = 2.24, 95% CI [1.84–2.71], *p* < 0.001), partially adjusted (OR = 1.49, 95% CI [1.22–1.80], *p* < 0.001), and completely adjusted (OR = 1.31, 95% CI [1.07–1.59], *p* = 0.007) models, dementia was the significant risk factor of NCH (Table 2, Table S6).

**Table 2:** Relationship between dementia and nontraumatic cerebral hemorrhage.

**Characteristic**	**Model 1**			**Model 2**			**Model 3**		
	**OR** ^1^	**95% CI** ^1^	***p*-value**	**OR** ^1^	**95% CI** ^1^	***p*-value**	**OR** ^1^	**95% CI** ^1^	***p*-value**
Dementia (Yes)	2.24	1.84, 2.71	<0.001	1.49	1.22, 1.80	<0.001	1.31	1.07, 1.59	0.007

**Notes.**

1 OR, Odds Ratio; CI, Confidence Interval.

Model 1: unadjusted.

Model 2: Age- and gender-based adjustments.

Model 3: Adjusted based on age, sex, hypertension, cerebral aneurysm, alcohol consumption, nicotine dependence, coagulation defects, CKMB, PTT, and PLT.

## Discussion

This study thoroughly examined the association between mental disorders and NCH based on MR analyses and observational studies. MR results suggested that only the genetically predicted dementia was causally related to NCH. The reverse MR analyses were conducted, given that mental disorders and NCH may reinforce each other. Genetically predicted NCH was not found to lead to the occurrence of dementia. Additionally, we did not observe any intermediate factors between dementia and NCH. Observational studies found that dementia was correlated with an increased risk of NCH, and this correlation persisted even after controlling for several confounding factors.

Current studies have demonstrated that the pathological mechanisms of NCH and mental disorders may overlap. Tryptophan metabolism is engaged in schizophrenia, bipolar disorder, major depressive disorder, ischemic stroke, NCH, Alzheimer’s disease, Parkinson’s disease, amyotrophic lateral sclerosis, and Huntington’s disease. Oxidative stress is also the common mechanism of both mental disorders and spontaneous NCH. It is worth noting that intraventricular extension of NCH is an important determinant of poor prognosis in NCH patients, potentially exacerbating secondary brain injury and worsening cognitive function. Although this study did not directly assess the impact of intraventricular extension, future research should consider the role of intraventricular extension in dementia-related NCH, as blood–brain barrier disruption and abnormal cerebral vascular regulation may render dementia patients more susceptible to intraventricular extension, thereby further worsening prognosis (Arboix et al., 2007; Puppala et al., 2023). Currently, many studies have explored the association of NCH with mental disorders. However, most of the studies assume that NCH occurs before mental disorders, and thus investigate mental disorders after NCH. Studies have found that acute cerebrovascular diseases increase the risks of depression and anxiety. However, the number of studies into whether mental disorders lead to NCH is limited. In this study, we explored the causal relationship between mental disorders and NCH through MR analyses with genetic variations as instrumental variables in MR. Such genetic predictors would be distributed in the population since alleles naturally randomize during meiosis. Therefore, theoretically, potential environmental confounding factors or medical issues would not have an impact on these genetic tools.

The MR analyses in this study provided genetic evidence supporting a potential causal relationship between genetically predicted dementia and NCH. Because genetic variants are fixed at conception and generally precede disease onset, the MR design is less susceptible to reverse causality than conventional observational studies. Moreover, by using genetic instruments, MR can also reduce bias from residual confounding, provided that the core instrumental variable assumptions are satisfied. Given the close clinical association between NCH and cognitive dysfunction, the observed relationship could still be questioned in terms of reverse causality. Therefore, reverse MR analyses were performed to determine whether genetically predicted NCH led to dementia. The results showed no reverse causal association between dementia and NCH. In addition, no significant mediating factors were found in the mediation analysis. This result may be attributed to multiple factors. First, the genetic contribution of the selected mediating variables may be low, necessitating further exploration of potential mediating factors. Additionally, the true mediating effect may be too weak or involve complex mechanisms such as nonlinear or time-varying effects, which the Mendelian randomization method has limited ability to identify (Richmond & Smith, 2022). An alternative explanation is that the mediation MR analysis may have been underpowered, particularly for mediators with relatively weak genetic instruments or limited GWAS sample sizes. Therefore, these negative results should be interpreted with caution (Burgess et al., 2017). Future studies should employ more sensitive analytical methods and consider more complex mediating mechanisms. However, during the process of identifying mediators, we found that genetically predicted dementia led to an increase in APO-B, APO-E, LDL, APO-B/APO-A1, TC, TG, and TJP-ZO-1, as well as a decrease in APO-A, HDL, and PLT. Among them, the association of apolipoproteins, lipid metabolites, and dementia has been proven (Palpatzis et al., 2022; Rasmussen, Luo & Frikke-Schmidt, 2024), but the number of studies on PLT and dementia is rather small, which is worth further exploration. Subsequently, similar correlations were also discovered in real human populations.

This study indicates that there is a causal association between dementia and NCH at the genetic level. In the real population, dementia is also correlated with NCH. However, there are still a few limitations in this study. Firstly, during the process of identifying mediators, univariate MR analyses utilized in this study may not demonstrate the direct effect of this biomarker on NCH, and the resulting causal effect may be induced by other targets in complex pathways. Secondly, although we screened intermediate factors as thoroughly as possible based on existing studies, there may still be potential mediators that have not been included due to research gaps and the lack of GWAS data. Thirdly, since the MIMIC database is retrospective, we cannot prospectively observe the occurrence of NCH. Thus, the observational study is a cross-sectional study, which limits the ability to capture temporality and infer causality. Future studies should employ prospective, multicenter cohort studies to validate the findings. In addition, genetically predicted dementia is proven to be causally related to NCH, but in addition to genetic factors, diet, lifestyle, and environmental factors may also contribute to the occurrence of dementia and NCH. Moreover, despite the potential association between specific sites of NCH and the development of vascular dementia, site-specific analyses were not possible due to data limitations in this study that prevented the acquisition of accurate hemorrhage localisation information. Further validation through prospective cohort studies combined with neuroimaging localisation is needed in the future. Furthermore, although this study adjusted for key confounding factors in the cross-sectional analysis, residual confounding factors, such as socioeconomic status and neuroimaging data, may still exist due to the limitations of the database variables. Moreover, for variables with a missing rate exceeding 25%, we analysed the missing values as a separate category. This may introduce missing data bias. Additionally, treating missing data as a separate category may introduce residual confounding. Finally, due to inconsistencies in population screening and confounding factor control standards among different GWAS data sources in the MRC-IEU database, population stratification bias and residual confounding bias may occur. Furthermore, in the MR analysis, there may be sample overlap between the exposure (FinnGen) and the outcome datasets. This overlap could introduce bias through a direct path between the instrumental variable and the outcome (not through the exposure). Furthermore, there is a potential sample overlap between the exposure and outcome datasets (both utilizing FinnGen data), which might introduce bias.

## Conclusion

This study investigated the association between psychiatric disorders and NCH through molecular genetic analyses and observational studies. Our findings provide genetic evidence supporting a potential causal association between genetically predicted dementia and NCH, and no evidence of reverse causality was found. However, no significant mediating pathways were identified, and the underlying biological mechanisms remain unclear. Therefore, this association should be interpreted with caution and requires validation in future studies using multicenter prospective cohort designs.

##  Supplemental Information

10.7717/peerj.21385/supp-1 Supplemental Information 1 Instrumental variables for psychosystemic disorders

10.7717/peerj.21385/supp-2 Supplemental Information 2 Results of Mendelian RandomizationResults of Reverse Mendelian Randomization

10.7717/peerj.21385/supp-3 Supplemental Information 3 Results of intermediary factor reverse Mendelian randomization analysis

10.7717/peerj.21385/supp-4 Supplemental Information 4 Results of inverse Mendelian randomization intermediary analysis

10.7717/peerj.21385/supp-5 Supplemental Information 5 Sensitivity analysis of multiple interpolation

10.7717/peerj.21385/supp-6 Supplemental Information 6 Results of logistic regression analysis

10.7717/peerj.21385/supp-7 Supplemental Information 7 References for Discussion

10.7717/peerj.21385/supp-8 Supplemental Information 8 STROBE checklist

## Abbreviations

MR: Mendelian randomization
NCH: non-traumatic cerebral hemorrhage
PAD: phobic anxiety disorder
ADHD: attention deficit hyperactivity disorder
IV: instrumental variables
CRP: C-reactive protein
TNF: tumor necrosis factor
ET: endothelin
ECE: endothelin-converting enzyme
HDL: lipoprotein
TC: total cholesterol
APO-A: apolipoprotein A
APO-B: apolipoprotein B
APO-E: apolipoprotein E
APO-B/APO-A1: apolipoprotein B/apolipoprotein A1
IL6: interleukin-6

## Additional Information and Declarations

### Data Availability

The following information was supplied regarding data availability: The MIMIC-IV dataset is available from PhysioNet at: - https://physionet.org/content/mimiciv/2.2/. Access to the MIMIC-IV dataset is restricted and is only available to credentialed users who have completed the required training and signed the required data use agreement. Specifically, users must complete the required CITI training course, “Data or Specimens Only Research,” and obtain access authorization through PhysioNet. The dataset is provided under the PhysioNet Credentialed Health Data License 1.5.0, and access requires agreement to the PhysioNet Credentialed Health Data Use Agreement 1.5.0. Only users who have signed the Data Use Agreement and received authorization are permitted to access the files. For this study, the required CITI training had been completed, and the CITI completion certificate was valid during the period of data access and analysis. The data is available at the Genome-Wide Association Study (GWAS) database: - GWAS ID: ebi-a-GCST012597, - GWAS ID: ebi-a-GCST90001390, - GWAS ID: ebi-a-GCST90038650, - GWAS ID: finn-b-F5_EATING, - GWAS ID: finn-b-F5_GENDER, - GWAS ID: finn-b-F5_HABIT, - GWAS ID: finn-b-F5_OCD, - GWAS ID: finn-b-F5_PERSMOOD, - GWAS ID: finn-b-F5_PHOBANX, - GWAS ID: finn-b-F5_PTSD, - GWAS ID: finn-b-F5_SLEEP, - GWAS ID: finn-b-F5_SOMATOFORM, - GWAS ID: finn-b-KRA_PSY_ANXIETY, - GWAS ID: finn-b-KRA_PSY_DEMENTIA, - GWAS ID: ieu-a-1185, - GWAS ID: ieu-b-5110.
