# Supplementary material for: Relationship between mental disorders and non-traumatic cerebral hemorrhage: cross-sectional analysis and mendelian randomization
Source: PeerJ. 2026 Jun 29;14:e21385. doi: 10.7717/peerj.21385 (PMC13326650; doi:10.7717/peerj.21385)
Supplement: Supplemental Information 2 [file peerj-14-21385-s002.docx]

**Supplementary table 2. Results of Mendelian Randomization.**

| **ID** | **Psychiatric disorders** | **Method** | **P-value** | **OR** | **Lower** | **Upper** | **Q** | **Q p-value** | **Egger intercept** | **P-value** |
| --- | --- | --- | --- | --- | --- | --- | --- | --- | --- | --- |
| ebi-a-GCST012597 | Attention deficit hyperactivity disorder | Inverse variance weighted | 0.5831 | 0.972 | 0.880 | 1.075 | 11.049 | 0.3537 | 0.0094 | 0.7466 |
|  |  | MR Egger | 0.6467 | 0.918 | 0.645 | 1.306 | 10.915 | 0.2816 |  |  |
|  |  | Weighted median | 0.9078 | 0.992 | 0.862 | 1.141 |  |  |  |  |
| ebi-a-GCST90001390 | Dementia with Lewy bodies | Inverse variance weighted | 0.1553 | 1.028 | 0.990 | 1.068 | 25.526 | 0.1111 | -0.0127 | 0.2867 |
|  |  | MR Egger | 0.1026 | 1.060 | 0.992 | 1.132 | 23.830 | 0.1241 |  |  |
|  |  | Weighted median | 0.0181 | 1.057 | 1.010 | 1.107 |  |  |  |  |
| ebi-a-GCST90038650 | Depression | Inverse variance weighted | 0.4793 | 3.120 | 0.133 | 72.995 | 30.914 | 0.3209 | 0.017 | 0.2253 |
|  |  | MR Egger | 0.3651 | 0.014 | 0.000 | 125.316 | 29.246 | 0.3491 |  |  |
|  |  | Weighted median | 0.5001 | 0.230 | 0.003 | 16.434 |  |  |  |  |
| finn-b-F5_EATING | Eating disorders | Inverse variance weighted | 0.1303 | 1.060 | 0.983 | 1.144 | 3.976 | 0.7826 | -0.0166 | 0.6838 |
|  |  | MR Egger | 0.4167 | 1.116 | 0.872 | 1.427 | 3.793 | 0.7047 |  |  |
|  |  | Weighted median | 0.1496 | 1.072 | 0.975 | 1.178 |  |  |  |  |
| finn-b-F5_GENDER | Gender identity disorders | Inverse variance weighted | 0.2715 | 0.99 | 0.973 | 1.008 | 5.104 | 0.6472 | -0.0128 | 0.5327 |
|  |  | MR Egger | 0.9321 | 0.999 | 0.968 | 1.030 | 4.666 | 0.5873 |  |  |
|  |  | Weighted median | 0.9840 | 1.000 | 0.977 | 1.023 |  |  |  |  |
| finn-b-F5_HABIT | Habit and impulse disorders | Inverse variance weighted | 0.6697 | 1.005 | 0.982 | 1.028 | 6.369 | 0.4974 | -0.0031 | 0.8318 |
|  |  | MR Egger | 0.6732 | 1.008 | 0.973 | 1.044 | 6.317 | 0.3886 |  |  |
|  |  | Weighted median | 0.9308 | 1.001 | 0.972 | 1.032 |  |  |  |  |
| finn-b-F5_OCD | Obsessive-compulsive disorder | Inverse variance weighted | 0.0765 | 1.042 | 0.996 | 1.090 | 5.776 | 0.8879 | 0.0095 | 0.6395 |
|  |  | MR Egger | 0.7339 | 1.018 | 0.919 | 1.128 | 5.543 | 0.8521 |  |  |
|  |  | Weighted median | 0.2431 | 1.036 | 0.976 | 1.100 |  |  |  |  |
| finn-b-F5_PERSMOOD | Persistent mood disorders | Inverse variance weighted | 0.5880 | 1.025 | 0.937 | 1.122 | 5.998 | 0.8155 | -0.0241 | 0.1841 |
|  |  | MR Egger | 0.1649 | 1.145 | 0.961 | 1.364 | 3.928 | 0.9161 |  |  |
|  |  | Weighted median | 0.5510 | 1.038 | 0.918 | 1.173 |  |  |  |  |
| finn-b-F5_PHOBANX | Phobic anxiety disorders | Inverse variance weighted | 0.9738 | 1.001 | 0.924 | 1.085 | 4.764 | 0.4453 | 0.0115 | 0.6262 |
|  |  | MR Egger | 0.7479 | 0.979 | 0.870 | 1.102 | 4.455 | 0.3479 |  |  |
|  |  | Weighted median | 0.3394 | 0.950 | 0.856 | 1.055 |  |  |  |  |
| finn-b-F5_PTSD | Post-traumatic stress disorder | Inverse variance weighted | 0.5541 | 1.017 | 0.961 | 1.077 | 11.372 | 0.2510 | 0.0382 | 0.1388 |
|  |  | MR Egger | 0.2581 | 0.926 | 0.818 | 1.048 | 8.501 | 0.3861 |  |  |
|  |  | Weighted median | 0.8501 | 1.007 | 0.937 | 1.082 |  |  |  |  |
| finn-b-F5_SLEEP | Sleep disorders | Inverse variance weighted | 0.5421 | 0.961 | 0.847 | 1.091 | 0.886 | 0.8289 | 0.0191 | 0.5516 |
|  |  | MR Egger | 0.4522 | 0.895 | 0.708 | 1.132 | 0.382 | 0.8260 |  |  |
|  |  | Weighted median | 0.4159 | 0.939 | 0.806 | 1.093 |  |  |  |  |
| finn-b-F5_SOMATOFORM | Somatoform disorder | Inverse variance weighted | 0.3817 | 0.971 | 0.910 | 1.037 | 17.327 | 0.3647 | 0.0083 | 0.5634 |
|  |  | MR Egger | 0.3748 | 0.934 | 0.806 | 1.081 | 16.933 | 0.3229 |  |  |
|  |  | Weighted median | 0.5576 | 0.973 | 0.888 | 1.066 |  |  |  |  |
| finn-b-KRA_PSY_ANXIETY | Anxiety disorders | Inverse variance weighted | 0.2326 | 0.917 | 0.796 | 1.057 | 17.174 | 0.6417 | 0.0179 | 0.2707 |
|  |  | MR Egger | 0.1393 | 0.776 | 0.562 | 1.071 | 15.887 | 0.6648 |  |  |
|  |  | Weighted median | 0.9281 | 1.009 | 0.828 | 1.230 |  |  |  |  |
| finn-b-KRA_PSY_DEMENTIA | Dementia | Inverse variance weighted | 1.157e-05 | 1.090 | 1.049 | 1.133 | 7.945 | 0.8922 | 0.0085 | 0.3819 |
|  |  | MR Egger | 0.0199 | 1.073 | 1.019 | 1.130 | 7.125 | 0.8956 |  |  |
|  |  | Weighted median | 0.0003 | 1.080 | 1.036 | 1.126 |  |  |  |  |
| ieu-a-1185 | Autism Spectrum Disorder | Inverse variance weighted | 0.7718 | 1.017 | 0.908 | 1.138 | 38.566 | 0.0882 | -0.0034 | 0.8251 |
|  |  | MR Egger | 0.7617 | 1.055 | 0.751 | 1.481 | 38.495 | 0.0704 |  |  |
|  |  | Weighted median | 0.5163 | 1.048 | 0.909 | 1.210 |  |  |  |  |
| ieu-b-5110 | Bipolar disorder | Inverse variance weighted | 0.2004 | 0.964 | 0.912 | 1.020 | 145.187 | 0.7409 | -0.0041 | 0.5399 |
|  |  | MR Egger | 0.7875 | 1.031 | 0.827 | 1.285 | 144.810 | 0.7295 |  |  |
|  |  | Weighted median | 0.6058 | 0.979 | 0.902 | 1.062 |  |  |  |  |
