# Supplementary material for: Relationship between mental disorders and non-traumatic cerebral hemorrhage: cross-sectional analysis and mendelian randomization
Source: PeerJ. 2026 Jun 29;14:e21385. doi: 10.7717/peerj.21385 (PMC13326650; doi:10.7717/peerj.21385)
Supplement: Supplemental Information 3 [file peerj-14-21385-s003.docx]

**Supplementary table 3. Results of inverse Mendelian randomization analysis**

| **ID** | **Psychiatric disorders** | **Method** | **P-value** | **OR** | **Lower** | **Upper** | **Q** | **Q p-value** | **Egger intercept** | **P-value** |
| --- | --- | --- | --- | --- | --- | --- | --- | --- | --- | --- |
| finn-b-KRA_PSY_DEMENTIA | Dementia | Inverse variance weighted | 0.2897 | 0.958 | 0.884 | 1.038 | 11.574 | 0.5628 | 0.0184 | 0.4125 |
|  |  | MR Egger | 0.2529 | 0.884 | 0.723 | 1.081 | 10.854 | 0.5415 |  |  |
|  |  | Weighted median | 0.1870 | 0.927 | 0.827 | 1.038 |  |  |  |  |
