# Supplementary material for: Relationship between mental disorders and non-traumatic cerebral hemorrhage: cross-sectional analysis and mendelian randomization
Source: PeerJ. 2026 Jun 29;14:e21385. doi: 10.7717/peerj.21385 (PMC13326650; doi:10.7717/peerj.21385)
Supplement: Supplemental Information 4 [file peerj-14-21385-s004.docx]

**Supplementary table 4. Results of intermediary analysis.**

| **ID** | **Intermediate factor** | **Method** | **Dementia vs. Intermediate factor** | | | | **Intermediate factor vs. Intracerebral hemorrhage** | | | | |
| --- | --- | --- | --- | --- | --- | --- | --- | --- | --- | --- | --- |
|  |  |  | **OR** | **Lower** | **Upper** | **P-value** | **nSNPs** | **OR** | **Lower** | **Upper** | **P-value** |
| bbj-a-22 | Fibrinogen | Weighted median | 1.004 | 0.971 | 1.037 | 0.8290 | 10 | 1.102 | 0.772 | 1.572 | 0.5929 |
|  |  | Inverse variance weighted | 1.011 | 0.969 | 1.054 | 0.6265 | 10 | 0.971 | 0.740 | 1.275 | 0.8341 |
|  |  | MR Egger | 0.990 | 0.939 | 1.044 | 0.7245 | 10 | 1.187 | 0.486 | 2.899 | 0.7168 |
| ebi-a-GCST004426 | TNF | Weighted median | 1.007 | 0.950 | 1.067 | 0.8262 | 5 | 1.018 | 0.762 | 1.358 | 0.9058 |
|  |  | Inverse variance weighted | 0.993 | 0.942 | 1.047 | 0.8074 | 5 | 1.040 | 0.837 | 1.291 | 0.7235 |
|  |  | MR Egger | 1.022 | 0.952 | 1.097 | 0.5601 | 5 | 0.779 | 0.557 | 1.089 | 0.2402 |
| ebi-a-GCST90002357 | PLT | MR Egger | 0.979 | 0.963 | 0.995 | 0.0210 | 718 | 1.115 | 0.933 | 1.332 | 0.2318 |
|  |  | Weighted median | 0.977 | 0.972 | 0.982 | 8.239e-20 | 718 | 1.148 | 0.972 | 1.356 | 0.1029 |
|  |  | Inverse variance weighted | 0.980 | 0.969 | 0.992 | 0.0007 | 718 | 1.081 | 0.975 | 1.198 | 0.1395 |
| ebi-a-GCST90002384 | HG | MR Egger | 0.991 | 0.977 | 1.006 | 0.2549 | 541 | 1.132 | 0.877 | 1.462 | 0.3412 |
|  |  | Weighted median | 0.995 | 0.990 | 1.001 | 0.1016 | 541 | 1.136 | 0.911 | 1.418 | 0.2579 |
|  |  | Inverse variance weighted | 0.999 | 0.988 | 1.011 | 0.8959 | 541 | 0.975 | 0.852 | 1.115 | 0.7081 |
| ebi-a-GCST90002412 | LDL | Weighted median | 1.016 | 1.001 | 1.031 | 0.0321 | 332 | 0.930 | 0.765 | 1.130 | 0.4636 |
|  |  | Inverse variance weighted | 1.207 | 1.137 | 1.282 | 8.743e-10 | 332 | 0.970 | 0.864 | 1.089 | 0.6028 |
|  |  | MR Egger | 1.247 | 1.151 | 1.352 | 0.0001 | 332 | 0.969 | 0.821 | 1.143 | 0.7070 |
| ebi-a-GCST90012005 | IL6 | MR Egger | 0.998 | 0.952 | 1.046 | 0.9351 | 12 | 1.225 | 0.747 | 2.008 | 0.4398 |
|  |  | Weighted median | 1.000 | 0.968 | 1.033 | 0.9950 | 12 | 1.196 | 0.909 | 1.574 | 0.2009 |
|  |  | Inverse variance weighted | 1.005 | 0.971 | 1.039 | 0.7948 | 12 | 1.155 | 0.912 | 1.464 | 0.2319 |
| ebi-a-GCST90013993 | APO-A | MR Egger | 0.903 | 0.869 | 0.938 | 0.0003 | 248 | 0.913 | 0.722 | 1.153 | 0.4440 |
|  |  | Weighted median | 0.923 | 0.909 | 0.938 | 1.031e-23 | 248 | 1.028 | 0.814 | 1.298 | 0.8196 |
|  |  | Inverse variance weighted | 0.925 | 0.897 | 0.954 | 8.723e-07 | 248 | 0.995 | 0.863 | 1.146 | 0.9395 |
| ebi-a-GCST90014002 | CRP | Weighted median | 1.007 | 0.992 | 1.022 | 0.3534 | 220 | 0.904 | 0.689 | 1.185 | 0.4633 |
|  |  | Inverse variance weighted | 0.959 | 0.907 | 1.015 | 0.1473 | 220 | 0.997 | 0.850 | 1.170 | 0.9734 |
|  |  | MR Egger | 0.986 | 0.889 | 1.095 | 0.8004 | 220 | 0.836 | 0.641 | 1.092 | 0.1906 |
| ebi-a-GCST90019454 | D-dimer | Weighted median | 0.990 | 0.956 | 1.024 | 0.5545 | 18 | 1.115 | 0.868 | 1.432 | 0.3947 |
|  |  | Inverse variance weighted | 1.000 | 0.970 | 1.030 | 0.9972 | 18 | 1.072 | 0.896 | 1.282 | 0.4483 |
|  |  | MR Egger | 0.976 | 0.937 | 1.016 | 0.2599 | 18 | 1.268 | 0.823 | 1.952 | 0.2974 |
| ebi-a-GCST90025953 | TC | MR Egger | 1.168 | 1.086 | 1.255 | 0.0136 | 193 | 1.042 | 0.831 | 1.307 | 0.7193 |
|  |  | Weighted median | 1.061 | 1.030 | 1.094 | 0.0001 | 193 | 1.022 | 0.813 | 1.284 | 0.8552 |
|  |  | Inverse variance weighted | 1.139 | 1.079 | 1.202 | 2.374e-06 | 193 | 1.034 | 0.882 | 1.212 | 0.6792 |
| ebi-a-GCST90092810 | APO-B/APO-A1 | Weighted median | 1.179 | 1.139 | 1.221 | 2.878e-20 | 66 | 1.068 | 0.842 | 1.354 | 0.5866 |
|  |  | Inverse variance weighted | 1.155 | 1.098 | 1.215 | 2.449e-08 | 66 | 1.042 | 0.887 | 1.225 | 0.6154 |
|  |  | MR Egger | 1.192 | 1.115 | 1.274 | 0.0002 | 66 | 1.140 | 0.862 | 1.507 | 0.3613 |
| ebi-a-GCST90095138 | tau-protein | MR Egger | 1.020 | 0.996 | 1.045 | 0.1239 | 16 | 0.921 | 0.834 | 1.016 | 0.1238 |
|  |  | Weighted median | 1.018 | 0.998 | 1.038 | 0.0777 | 16 | 0.968 | 0.871 | 1.074 | 0.5375 |
|  |  | Inverse variance weighted | 1.014 | 0.996 | 1.032 | 0.1172 | 16 | 0.969 | 0.885 | 1.062 | 0.5025 |
| ieu-b-108 | APO-B | MR Egger | 1.262 | 1.159 | 1.374 | 0.0001 | 184 | 1.203 | 0.990 | 1.461 | 0.0643 |
|  |  | Inverse variance weighted | 1.216 | 1.140 | 1.296 | 2.606e-09 | 184 | 0.953 | 0.821 | 1.105 | 0.5223 |
|  |  | Weighted median | 1.018 | 1.001 | 1.034 | 0.0355 | 184 | 1.263 | 1.014 | 1.573 | 0.0372 |
| ieu-b-109 | HDL | Weighted median | 0.937 | 0.928 | 0.946 | 5.980e-39 | 326 | 0.993 | 0.799 | 1.234 | 0.9477 |
|  |  | MR Egger | 0.925 | 0.897 | 0.955 | 0.0003 | 326 | 1.059 | 0.875 | 1.281 | 0.5562 |
|  |  | Inverse variance weighted | 0.949 | 0.924 | 0.975 | 0.0002 | 326 | 0.923 | 0.815 | 1.047 | 0.2130 |
| ieu-b-111 | TG | Inverse variance weighted | 1.043 | 1.025 | 1.060 | 9.648e-07 | 284 | 0.984 | 0.857 | 1.130 | 0.8190 |
|  |  | MR Egger | 1.063 | 1.044 | 1.082 | 1.733e-05 | 284 | 0.962 | 0.784 | 1.181 | 0.7145 |
|  |  | Weighted median | 1.051 | 1.041 | 1.062 | 2.576e-23 | 284 | 0.882 | 0.703 | 1.108 | 0.2810 |
| prot-a-132 | APO-E | Inverse variance weighted | 1.212 | 1.138 | 1.291 | 2.071e-09 | 14 | 1.045 | 0.930 | 1.174 | 0.4633 |
|  |  | MR Egger | 1.253 | 1.152 | 1.363 | 0.0002 | 14 | 1.123 | 0.870 | 1.451 | 0.3908 |
|  |  | Weighted median | 1.250 | 1.173 | 1.332 | 5.751e-12 | 14 | 0.978 | 0.830 | 1.152 | 0.7904 |
| prot-a-242 | BDNF | Weighted median | 0.951 | 0.897 | 1.009 | 0.0995 | 14 | 0.911 | 0.767 | 1.083 | 0.2912 |
|  |  | MR Egger | 0.950 | 0.883 | 1.021 | 0.1876 | 14 | 0.959 | 0.657 | 1.400 | 0.8317 |
|  |  | Inverse variance weighted | 0.953 | 0.903 | 1.006 | 0.0787 | 14 | 0.941 | 0.829 | 1.069 | 0.3483 |
| prot-a-2983 | TJP-ZO-1 | MR Egger | 1.236 | 1.150 | 1.328 | 6.807e-05 | 10 | 1.019 | 0.751 | 1.383 | 0.9073 |
|  |  | Inverse variance weighted | 1.166 | 1.099 | 1.238 | 4.533e-07 | 10 | 1.012 | 0.867 | 1.182 | 0.8772 |
|  |  | Weighted median | 1.223 | 1.147 | 1.304 | 8.699e-10 | 10 | 0.991 | 0.831 | 1.181 | 0.9168 |
| prot-a-498 | CDNF | Inverse variance weighted | 0.967 | 0.916 | 1.020 | 0.2153 | 16 | 0.959 | 0.872 | 1.055 | 0.3912 |
|  |  | MR Egger | 0.958 | 0.891 | 1.030 | 0.2657 | 16 | 1.151 | 0.946 | 1.402 | 0.1819 |
|  |  | Weighted median | 0.969 | 0.915 | 1.026 | 0.2828 | 16 | 0.970 | 0.843 | 1.116 | 0.6703 |
| prot-a-883 | ECE | Weighted median | 0.967 | 0.909 | 1.028 | 0.2824 | 15 | 0.982 | 0.870 | 1.108 | 0.7669 |
|  |  | MR Egger | 0.970 | 0.902 | 1.043 | 0.4206 | 15 | 0.899 | 0.742 | 1.089 | 0.2957 |
|  |  | Inverse variance weighted | 0.966 | 0.915 | 1.019 | 0.2011 | 15 | 1.001 | 0.912 | 1.098 | 0.9859 |
| prot-a-892 | ET | Weighted median | 0.950 | 0.895 | 1.009 | 0.0945 | 3 | 0.966 | 0.842 | 1.109 | 0.6235 |
|  |  | MR Egger | 0.925 | 0.848 | 1.009 | 0.1035 | 3 | 0.905 | 0.584 | 1.400 | 0.7310 |
|  |  | Inverse variance weighted | 0.943 | 0.885 | 1.004 | 0.0671 | 3 | 0.978 | 0.855 | 1.117 | 0.7393 |
