# Supplementary material for: Relationship between mental disorders and non-traumatic cerebral hemorrhage: cross-sectional analysis and mendelian randomization
Source: PeerJ. 2026 Jun 29;14:e21385. doi: 10.7717/peerj.21385 (PMC13326650; doi:10.7717/peerj.21385)
Supplement: Supplemental Information 5 [file peerj-14-21385-s005.docx]

**Supplementary table 5. Sensitivity analysis of multiple interpolation.**

| **Characteristic** | **Overall** N = 361,466*^1^* | **After MP** N = 180,733*^1^* | **Before MP** N = 180,733*^1^* | **p-value***^2,3^* |
| --- | --- | --- | --- | --- |
| Platelets | 242 [195, 297] | 242 [195, 297] | 242 [195, 297] | 0.9 |
| PT | 12.3 [11.3, 13.5] | 12.3 [11.3, 13.5] | 12.3 [11.3, 13.5] | 0.2 |
| PTT | 29 [26, 32] | 29 [26, 32] | 29 [26, 32] | 0.10 |
| *^1^* Median [Q1, Q3]  *^2^* Wilcoxon rank sum test  *^3^* *p<0.05; **p<0.01; ***p<0.001 | | | | |
