# Supplementary material for: Relationship between mental disorders and non-traumatic cerebral hemorrhage: cross-sectional analysis and mendelian randomization
Source: PeerJ. 2026 Jun 29;14:e21385. doi: 10.7717/peerj.21385 (PMC13326650; doi:10.7717/peerj.21385)
Supplement: Supplemental Information 6 [file peerj-14-21385-s006.docx]

**Supplementary table 6. Results of logistic regression analysis**

| **Characteristic** | **OR***^1^* | **95% CI***^1^* | **p-value** |
| --- | --- | --- | --- |
| **Model 1** | | | |
| Dementia |  |  |  |
| No | — | — |  |
| Yes | 2.24 | 1.84, 2.71 | <0.001 |
| **Model 2** | | | |
| Dementia |  |  |  |
| No | — | — |  |
| Yes | 1.49 | 1.22, 1.80 | <0.001 |
| Gender |  |  |  |
| Female | — | — |  |
| Male | 1.2 | 1.12, 1.28 | <0.001 |
| Age |  |  |  |
| <=60 years | — | — |  |
| > years | 2.61 | 2.43, 2.81 | <0.001 |
| **Model 3** | | | |
| Dementia |  |  |  |
| No | — | — |  |
| Yes | 1.31 | 1.07, 1.59 | 0.007 |
| Gender |  |  |  |
| Female | — | — |  |
| Male | 1.09 | 1.02, 1.17 | 0.015 |
| Age |  |  |  |
| <=60 years | — | — |  |
| > 60 years | 1.68 | 1.55, 1.81 | <0.001 |
| Alcohol |  |  |  |
| No | — | — |  |
| Yes | 1.11 | 0.94, 1.30 | 0.2 |
| Cerebral aneurysm |  |  |  |
| No | — | — |  |
| Yes | 5.13 | 4.20, 6.22 | <0.001 |
| Coagulation deficiencies |  |  |  |
| No | — | — |  |
| Yes | 1.16 | 1.02, 1.31 | 0.018 |
| Hypertension |  |  |  |
| No | — | — |  |
| Yes | 2.08 | 1.93, 2.23 | <0.001 |
| Nicotine dependence |  |  |  |
| No | — | — |  |
| Yes | 0.97 | 0.80, 1.16 | 0.7 |
| Respiratory failure |  |  |  |
| No | — | — |  |
| Yes | 4.04 | 3.65, 4.45 | <0.001 |
| Platelets | 1 | 1.00, 1.00 | <0.001 |
| PTT | 0.97 | 0.96, 0.97 | <0.001 |
