# Supplementary material for: Relationship between mental disorders and non-traumatic cerebral hemorrhage: cross-sectional analysis and mendelian randomization
Source: PeerJ. 2026 Jun 29;14:e21385. doi: 10.7717/peerj.21385 (PMC13326650; doi:10.7717/peerj.21385)
Supplement: Supplemental Information 7 [file peerj-14-21385-s007.docx]

**Supplementary 6 References for Discussion**

1. Schwarcz R, Bruno JP, Muchowski PJ, Wu H-Q. Kynurenines in the mammalian brain: when physiology meets pathology. Nature Reviews Neuroscience. 2012;13(7):465-77
2. Sellgren C, Kegel M, Bergen S, Ekman C, Olsson S, Larsson M, et al. A genome-wide association study of kynurenic acid in cerebrospinal fluid: implications for psychosis and cognitive impairment in bipolar disorder. Molecular psychiatry. 2016;21(10):1342-50
3. Réus GZ, Jansen K, Titus S, Carvalho AF, Gabbay V, Quevedo J. Kynurenine pathway dysfunction in the pathophysiology and treatment of depression: Evidences from animal and human studies. Journal of psychiatric research. 2015;68:316-28
4. Isabel Cuartero M, de la Parra J, Garcia-Culebras A, Ballesteros I, Lizasoain I. The kynurenine pathway in the acute and chronic phases of cerebral ischemia. Current pharmaceutical design. 2016;22(8):1060-73
5. Thilo F, Suess O, Liu Y, Tepel M. Decreased expression of transient receptor potential channels in cerebral vascular tissue from patients after hypertensive intracerebral hemorrhage. Clinical and experimental hypertension. 2011;33(8):533-7
6. Bohár Z, Toldi J, Fülöp F, Vécsei L. Changing the face of kynurenines and neurotoxicity: therapeutic considerations. International journal of molecular sciences. 2015;16(5):9772-93
7. Tutakhail A, Boulet L, Khabil S, Nazari QA, Hamid H, Coudoré F. Neuropathology of kynurenine pathway of tryptophan metabolism. Current pharmacology reports. 2020;6:8-23
8. Iłżecka J, Kocki T, Stelmasiak Z, Turski W. Endogenous protectant kynurenic acid in amyotrophic lateral sclerosis. Acta neurologica scandinavica. 2003;107(6):412-8
9. Veres G, Molnár M, Zádori D, Szentirmai M, Szalárdy L, Török R, et al. Central nervous system-specific alterations in the tryptophan metabolism in the 3-nitropropionic acid model of Huntington's disease. Pharmacology Biochemistry and Behavior. 2015;132:115-24
10. Thangameeran SIM, Wang P-K, Liew H-K, Pang C-Y. Influence of Alcohol on Intracerebral Hemorrhage: From Oxidative Stress to Glial Cell Activation. Life. 2024;14(3):311
11. Kim Y, Vadodaria KC, Lenkei Z, Kato T, Gage FH, Marchetto MC, et al. Mitochondria, metabolism, and redox mechanisms in psychiatric disorders. Antioxidants & redox signaling. 2019;31(4):275-317
12. Song T, Song X, Zhu C, Patrick R, Skurla M, Santangelo I, et al. Mitochondrial dysfunction, oxidative stress, neuroinflammation, and metabolic alterations in the progression of Alzheimer’s disease: A meta-analysis of in vivo magnetic resonance spectroscopy studies. Ageing research reviews. 2021;72:101503
13. Zhu L, Yu C, Chang Y, Sun S, Sun Z. Serum Cystatin C is Associated with Depression After Intracerebral Hemorrhage. Neuropsychiatric Disease and Treatment. 2023:1117-26
14. Sallinen H, Sairanen T, Strbian D. Quality of life and depression 3 months after intracerebral hemorrhage. Brain and Behavior. 2019;9(5):e01270
15. An P, Zhao X-C, Liu M-J, You Y-Q, Li J-Y. Gender-based differences in neuroprotective effects of hydrogen gas against intracerebral hemorrhage-induced depression. Neurochemistry International. 2022;153:105276
16. Koivunen RJ, Harno H, Tatlisumak T, Putaala J. Depression, anxiety, and cognitive functioning after intracerebral hemorrhage. Acta neurologica Scandinavica. 2015;132(3):179-84
17. Scopelliti G, Casolla B, Boulouis G, Kuchcinski G, Moulin S, Leys D, et al. Long-term anxiety in spontaneous intracerebral hemorrhage survivors. International Journal of Stroke. 2022;17(10):1093-9
18. Zheng J, Baird D, Borges M-C, Bowden J, Hemani G, Haycock P, et al. Recent developments in Mendelian randomization studies. Current epidemiology reports. 2017;4:330-45
19. Davies NM, Holmes MV, Smith GD. Reading Mendelian randomisation studies: a guide, glossary, and checklist for clinicians. bmj. 2018;362
20. Raulin A-C, Doss SV, Trottier ZA, Ikezu TC, Bu G, Liu C-C. ApoE in Alzheimer’s disease: Pathophysiology and therapeutic strategies. Molecular neurodegeneration. 2022;17(1):72
21. Martens YA, Zhao N, Liu C-C, Kanekiyo T, Yang AJ, Goate AM, et al. ApoE Cascade Hypothesis in the pathogenesis of Alzheimer’s disease and related dementias. Neuron. 2022;110(8):1304-17
